# Navigating the AI frontiers in cardiovascular research: a bibliometric exploration and topic modeling

**DOI:** 10.3389/fcvm.2023.1308668

**Published:** 2024-01-03

**Authors:** Kirubel Biruk Shiferaw, Payam Wali, Dagmar Waltemath, Atinkut Alamirrew Zeleke

**Affiliations:** Department of Medical Informatics, Institute for Community Medicine, University Medicine Greifswald, Greifswald, Germany

**Keywords:** cardiovascular, scientometric, bibliometric, topic modeling, artificial intelligence, machine learning

## Abstract

Artificial intelligence (AI) has emerged as a promising field in cardiovascular disease (CVD) research, offering innovative approaches to enhance diagnosis, treatment, and patient outcomes. In this study, we conducted bibliometric analysis combined with topic modeling to provide a comprehensive overview of the AI research landscape in CVD. Our analysis included 23,846 studies from Web of Science and PubMed, capturing the latest advancements and trends in this rapidly evolving field. By employing LDA (Latent Dirichlet Allocation) we identified key research themes, trends, and collaborations within the AI-CVD domain.

The findings revealed the exponential growth of AI-related research in CVD, underscoring its immense potential to revolutionize cardiovascular healthcare. The annual scientific publication of machine learning papers in CVD increases continuously and significantly since 2016, with an overall annual growth rate of 22.8%. Almost half (46.2%) of the growth happened in the last 5 years. USA, China, India, UK and Korea were the top five productive countries in number of publications. UK, Germany and Australia were the most collaborative countries with a multiple country publication (MCP) value of 42.8%, 40.3% and 40.0% respectively. We observed the emergence of twenty-two distinct research topics, including “stroke and robotic rehabilitation therapy,” “robotic-assisted cardiac surgery,” and “cardiac image analysis,” which persisted as major topics throughout the years. Other topics, such as “retinal image analysis and CVD” and “biomarker and wearable signal analyses,” have recently emerged as dominant areas of research in cardiovascular medicine.

Convolutional neural network appears to be the most mentioned algorithm followed by LSTM (Long Short-Term Memory) and KNN (K-Nearest Neighbours). This indicates that the future direction of AI cardiovascular research is predominantly directing toward neural networks and image analysis.

As AI continues to shape the landscape of CVD research, our study serves as a comprehensive guide for researchers, practitioners, and policymakers, providing valuable insights into the current state of AI in CVD research. This study offers a deep understanding of research trends and paves the way for future directions to maximiz the potential of AI to effectively combat cardiovascular diseases.

## Introduction

The application of advanced big-data analysis appears to shape the future of healthcare (1–3). Artificial intelligence (AI) has become a common tool for researchers and practitioners in several medical contexts (4). One medical condition where AI is extensively used is cardiovascular disease (CVD) (5). CVD is one of the leading causes of death in the world (33% of all deaths) and reason for more than 60 million potential years of life lost in Europe (6). A steadily increasing number of studies are conducted to understand the complex and dynamic nature of CVD, ascertaining the relevance of AI for risk, treatment and event/outcome prediction/classification. Publications in peer reviewed academic journals suggesting a customized machine learning (ML), deep learning (DL) and natural language processing (NLP) models for diagnosis and prognosis of CVD is increasing (7).

Several studies have explored the increasing application of AI in CVD, including early detection of stroke and heart disease (7). The study depicted a sharp increase in number of publications on the topic of applied AI and pointed out the increased use of robotics in diagnostics and stroke rehabilitation. Similarly, a systematic review by Arshia et al., showed the exponentially growing number of publications regarding the application of AI and ML in vascular surgery (8). The study analysed 249 literatures from three databases (MEDLINE, Embase and Ovid HeathStar) and indicated that carotid artery disease, peripheral arterial disease and abdominal aortic aneurysm are the most dominant topics in which AI and ML methods are highly applied in the vascular surgery domain. The review also showed that neural networks and support vector machines were the most frequently applied methods. According to Ginette et al. the overall trend for wearable technologies in cardiovascular disease has increased, and the application of AI became prominent since 2020 (9). The study also commented on the significant imbalance in scientific contribution from low income countries.

Other studies have explored the use of AI in specific aspects of cerebrovascular and heart diseases. For example, in a review by Andrew et al., the potential advancement and benefits of decision support systems developed to aid clinicians in selecting the most appropriate treatment strategy for acute ischemic stroke was discussed (10). Despite these efforts, a comprehensive analysis of the general research landscape on the application of AI in managing cardiovascular diseases would benefit in shaping the direction of research and signalling the emerging topics and methods in applying AI in cardiovascular medicine.

Synthesis of the vastly increasing scientific evidence in this field is needed to identify the trends and topics that have been studied over the years. In this context, bibliometric analysis is a suitable research methodology as it enables researchers to explore the current trend in a particular research area using citation information (11). It mainly provides an overview of who is doing what, where, with whom and the intensity of collaboration across countries, authors and affiliations. Additionally, topic modeling helps in discovering patterns of word use and connecting documents with similar patterns to identify dominant topics from a large corpus of text (12).

Undoubtedly, the application of AI methods in CVD has evolved over time, influenced by the advancement in computer processing power and rapidly evolving research in data science. Therefore, by identifying the key trends and research themes in CVD and AI, this study aims to guide future research and development efforts in the field of in AI-enhanced healthcare for cardiovascular diseases.

## Methods

### Search strategy

Keywords from previous literature and the 2019 online version of the International Statistical Classification of Diseases and Related Health Problems 10th Revision (ICD-10) have been used to scan for all CVD related diseases (13, 14). Medical subheadings, mixing keywords with subject headings, truncated keywords and controlled vocabularies were applied in iterative manner. We implemented a systematic approach to make sure the included papers are relevant. The search was commenced by two independent reviewers and crosschecked to ensure consistency in the search result. Afterward, we removed duplicates and curated for papers with no citation information.. The search was conducted on PubMed and Web of Science (WoS) by limiting the year from 2000 to 2022. Any discrepancies in the search results were discussed and resolved.

### Screening strategy

**Figure 1:**
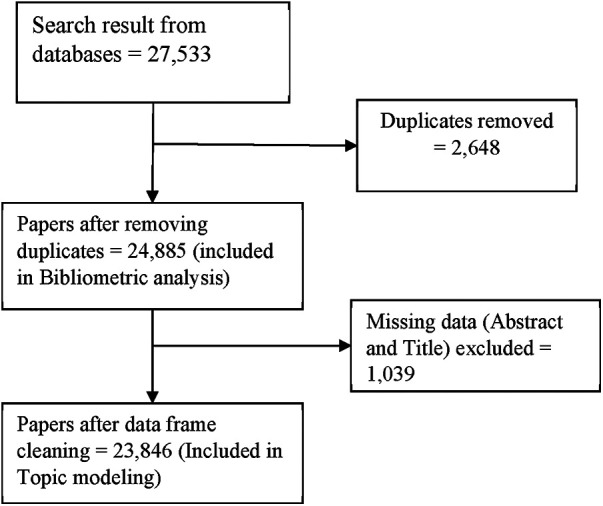
Search result flowchart. The search results of keywords associated with CVD and AI.

Journal articles, preceding papers, reviews, conference abstracts, early access and book chapters were included. The search was limited to English language, human studies and studies with abstract. The search was further restricted to studies that had applied AI/ML as a major intervention in cardiovascular medicine. After the search by the two independent reviewers (KBS and PW), duplicates were removed using Endnote. The detailed search results are presented in Figure 1.

## Analysis

Descriptive analysis, bibliographic network visualizations and network analysis were performed using the Bibliometrix package in R and the VOS viewer (15, 16). The trend in publication and collaboration network will show the number of published studies related to AI and CVD, who is contributing more, who is collaborating most, and where those studies are from.

Topic modeling is an unsupervised machine learning algorithm to extract hidden topics or semantic structure from a corpus of text. It will help us identify key words and dominant topics associated with AI and CVD. The method assumes that all text in a specific document is composed of different topics and every topic is composed of related words (12). The most common approaches of topic modeling are: Latent semantic analysis (LSA), probabilistic Latent Semantic Analysis (pLSA), and Latent Dirichlet allocation (LDA). LSA is a statistical method to extract and represent the contextual meaning of words in a large volume of unstructured corpus of text based on their proximity in semantic space (17, 18). pLSA is another probabilistic statistical approach that aims to differentiate and identify meanings of words in different contexts (19).

**Figure 2:**
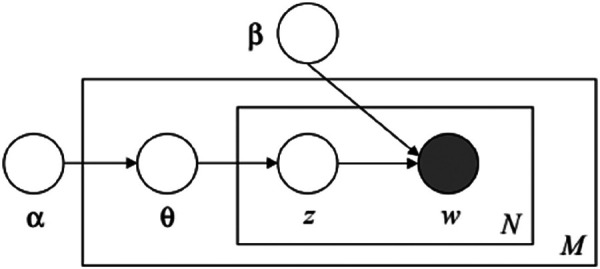
Graphical representation of LDA model. The boxes represent replicates. The outer plate represents total number of documents (M), while the inner plate represents the repeated choice of topics (z) and words (w) within a document (N). α, represents” topic distribution per document”; β, represents “word distribution per topic”; θ, represents “topic distribution per document M”.

LDA considers documents as a random mixture of latent topics and topics as a dominant distribution of words (17). It is the most appropriate model for the research question addressed in this work. Figure 2 illustrates the detail of LDA model representation demonstrated by Blei et al. (17).

We applied the data pre-processing steps presented in Table 1 prior to the topic modeling.

**Table 1:** Data cleaning and pre-processing steps.

Step	Description
1. Tokenization	Tokenization is breaking down texts to subunits of words called tokens (20)
2. Punctuation and special character removal	All irrelevant characters like question marks, coma and semicolon are removed from the text. Furthermore, to avoid repeated representation of tokens, the text was converted to small letters
3. Stop word removal	Stop words are words used in the text for grammatical and semantic purposes but not directly related to the topic in the document. Words such as “is”, “are”, “you” “the”, should be removed from the corpus in order to receive a more representative topic. Additionally, most common words and most rare words are considered stop words to optimize the result. Therefore, the set of stop words was extended with words like “background”, “method”, or “results” to avoid representation of non-relevant words
4. Lemmatization	Lemmatization is limiting a word to its original root word (21). The machine should recognize both “predicting” and “prediction” as “predict”, not as two different words
5. Bigrams and trigrams	Bigrams and trigrams are words that frequently occur together in pairs or in triples, respectively. For example, sometimes phrases like “convolutional neural network” appear together, and the model should read this as a single word (group) not as three individual words

For the topic modeling, we analyzed publication's title, abstract and author keywords from 2010 to 2022. The topics were clustered and visualized using intertopic distance map and t- stochastic neighbor embeding t-SNE.

## Results

The search resulted in a total number of 27,533 studies. After removing duplicates, 24,885 studies were considered for further bibliometric analysis. For subsequent topic modeling analysis, 23,846 studies were considered after removing 1,039 studies with missing abstract or title (see Figure 1).

**Figure 3:**
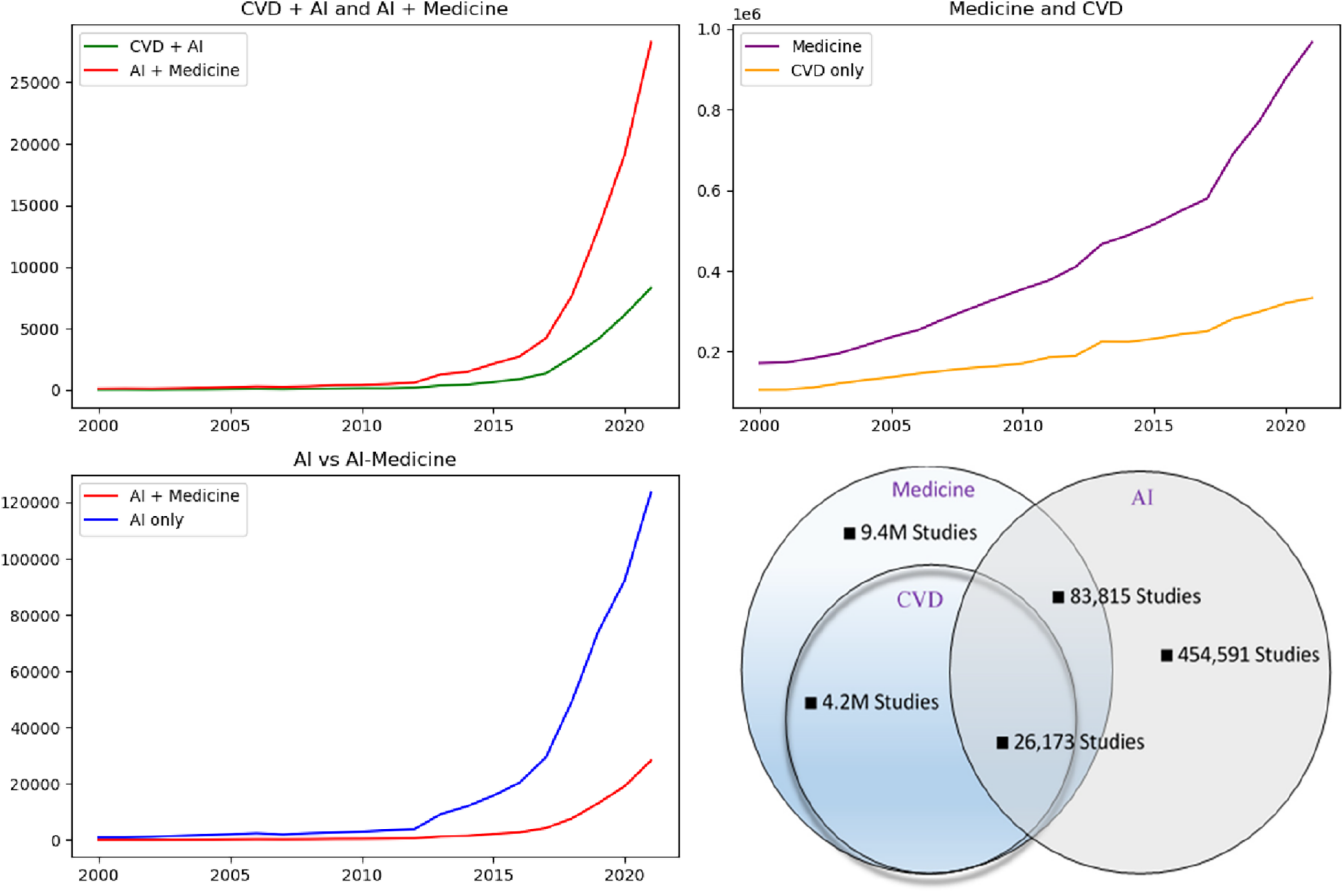
Annual publication trend. Studies related to the application of AI in cardiovascular disease and medicine since 2000.

More than 60% of the publications were journal articles followed by proceeding papers (19%). The annual scientific production of machine learning papers in CVD increases continuously and significantly since 2016, with an overall annual growth rate of 22.8%. Almost half (46.2%) of the growth happened in the last 5 years. A significantly higher number of studies with a focus on the application of AI in CVD is indexed in WoS than in PubMed (Figure 3). To have a clear insight, we also compared the results with AI-medicine search result and the comparison showed that AI application in CVD domain shares a significant portion (31.2%) of AI studies in medicine.

We also saw that USA, China, India, UK and Korea were the top five productive countries in number of publications. UK, Germany and Australia were the most collaborative countries with a multiple country publication (MCP) value of 42.8%, 40.3% and 40.0% respectively (Table 2). The country of first author's affiliation is considered as corresponding author's country and the MCP ratio is calculated using the publications authored by authors from multiple countries divided by the total publication record of the country.

**Table 2:** Most productive corresponding author's countries and collaboration ratio.

Country	Number of publications		SCP		MCP		MCP_ratio (%)	
	WoS	PubMed	WoS	PubMed	WoS	PubMed	WoS	PubMed
USA	4,470	1,543	3,382	1,354	1,088	189	24.3%	12.3%
China	3,942	722	2,965	577	977	145	24.8%	20.1%
India	1,158	124	1,007	100	151	24	13.0%	19.4%
United Kingdom	906	72	518	55	388	17	42.8%	23.6%
Korea	745	276	608	245	137	31	18.4%	11.2%
Germany	601	229	359	185	242	44	40.3%	19.2%
Canada	513	161	335	128	178	33	34.7%	20.5%
Italy	488	288	332	236	156	52	32.0%	18.1%
Japan	479	255	396	195	83	30	17.3%	13.3%
Australia	408	90	245	66	163	24	40.0%	26.7%
France	368	162	237	136	131	26	35.6%	16.1%
Spain	368	92	246	71	122	21	33.2%	22.8%
Netherlands	284	127	172	91	112	36	39.4%	28.4%
Brazil	204	49	134	35	70	14	34.3%	28.6%
Turkey	201	120	172	110	29	10	14.4%	8.3%

SCP, single country publication (all authors belongs to the same country); MCP, multiple country publication (the authors are from different countries).

**Figure 4:**
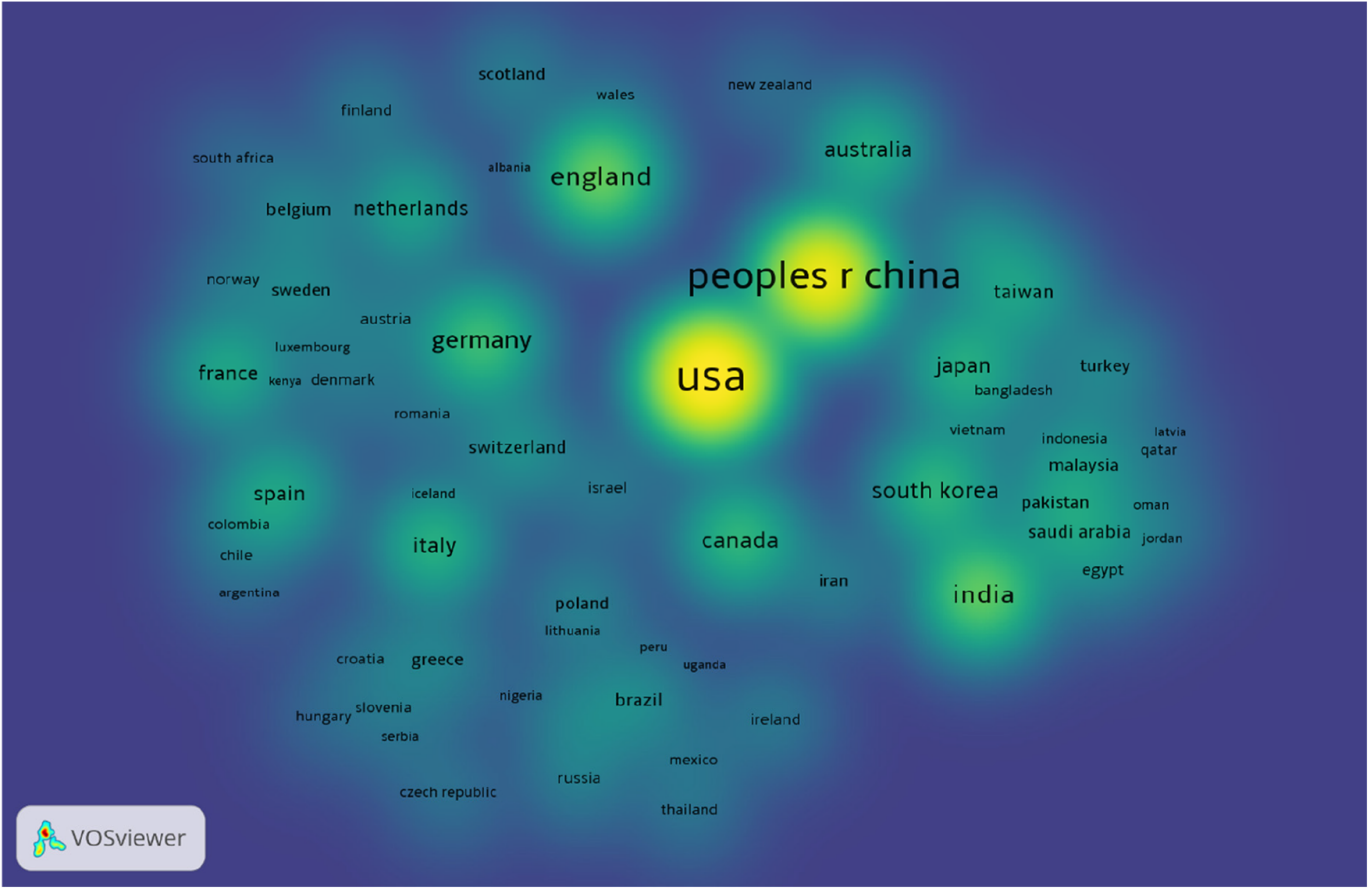
Density map of publications in the area of AI in CVD context. The density size and color show the intensity of contribution.

The density map in Figure 4 showed that most of the studies conducted in this area of research arises from developed countries and it is also important to note the population size in interpreting this result. The figure only shows the number of publications and not the proportion per population size. Yet this can be an important input to understand the overall publication size in this domain of research.

**Figure 5:**
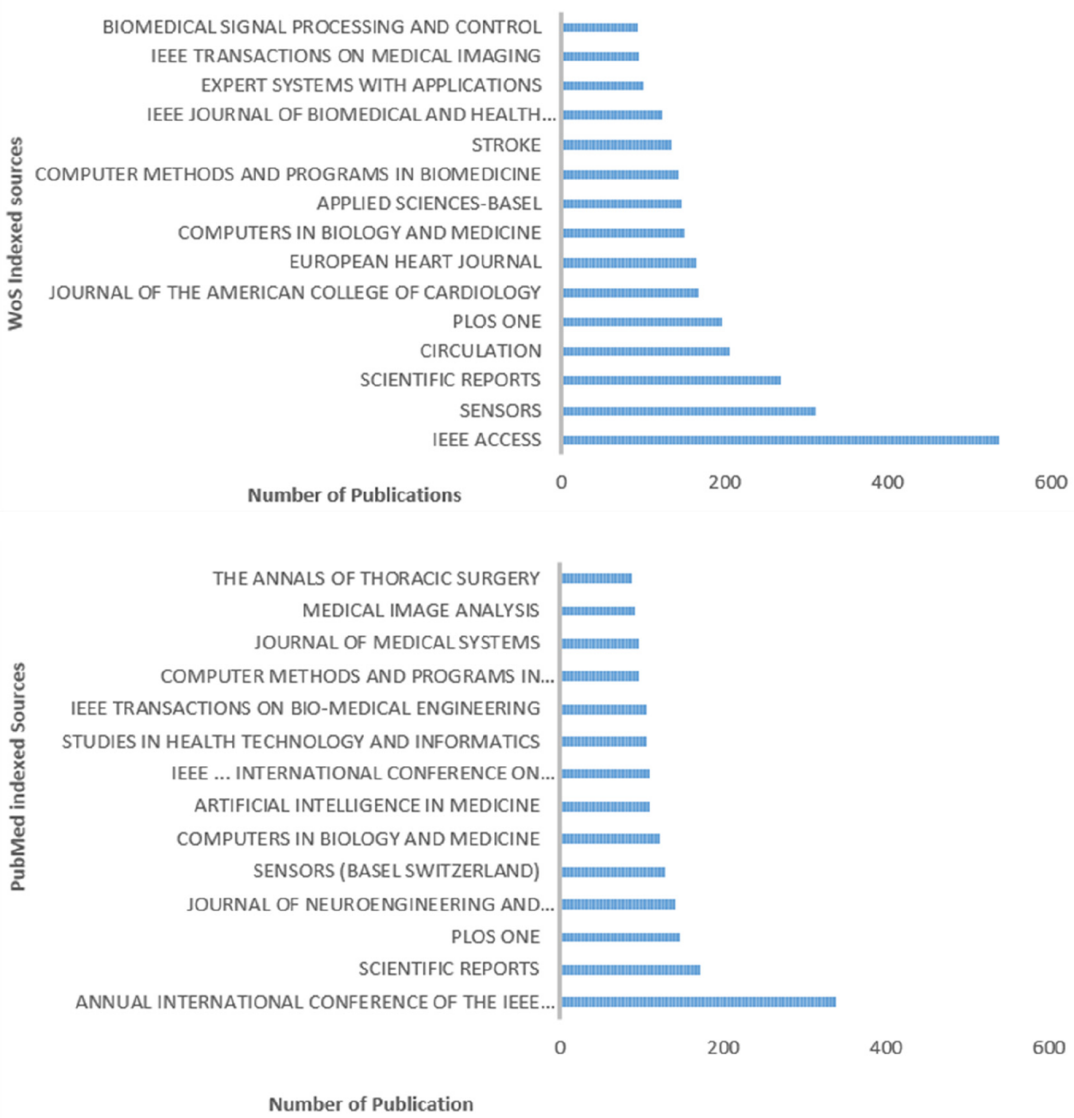
Most relevant sources indexed in WoS and PubMed. The graph shows where most of the publications associated with CVD and AI are published between 2000 and 2022.

IEEE access, IEEE engineering in medicine and biology (annual conference), Scientific reports, PlosOne and sensors were the top five sources indexed in WoS (Figure 5).

## Results for the topic modeling

After cleaning the list of studies, the abstracts from each study was extracted for further analysis. The number of topic was identified by using coherence (22). The coherence score calculates how words in a particular topic are similar to each other based on the calculated word co-occurrence, normalized pointwise mutual information (NPMI) and the cosine similarity (23).

**Figure 6:**
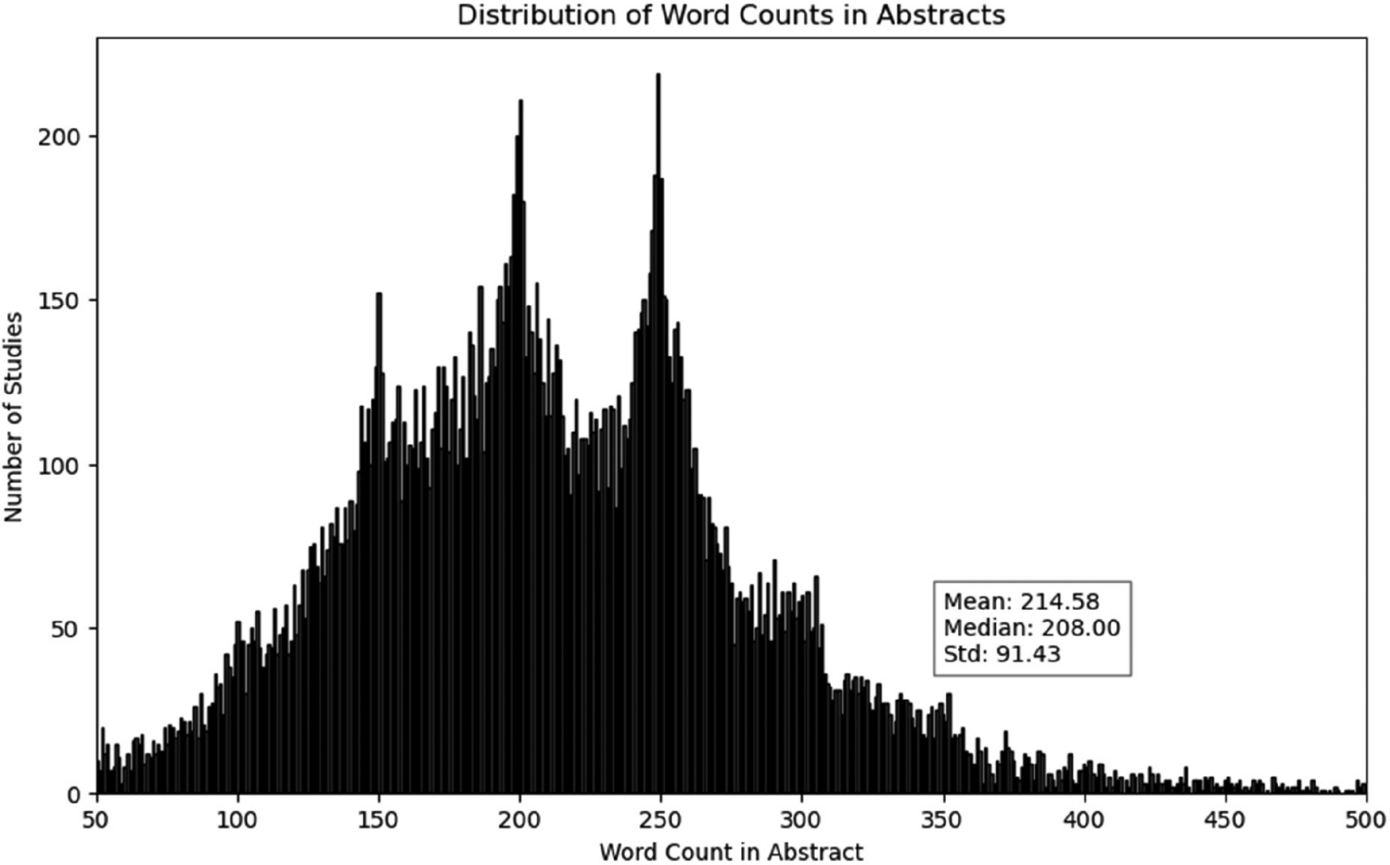
Distribution of word count in documents of publications in 2010–2022. The y-axis represents the number of studies and the x-axis represents the word counts in the abstract. Most abstracts have a word range between 100 and 300 words.

The distribution of word counts across documents is assessed to visualize the patterns in the data and the shape of word distribution among abstracts. It is important to assess the word distribution because if the distribution is heavily skewed to the left, with a large proportion of documents having a low word count, it may indicate that many of the documents are short or that the data is not well-suited for LDA analysis. The average word count per abstract were 215 words with standard deviation of 92 words (Figure 6). This imply that that the average word count in the abstracts is more or less representative (meaning, the tail effect is reduced considering that most journals limit the abstract to 350 words).

**Figure 7:**
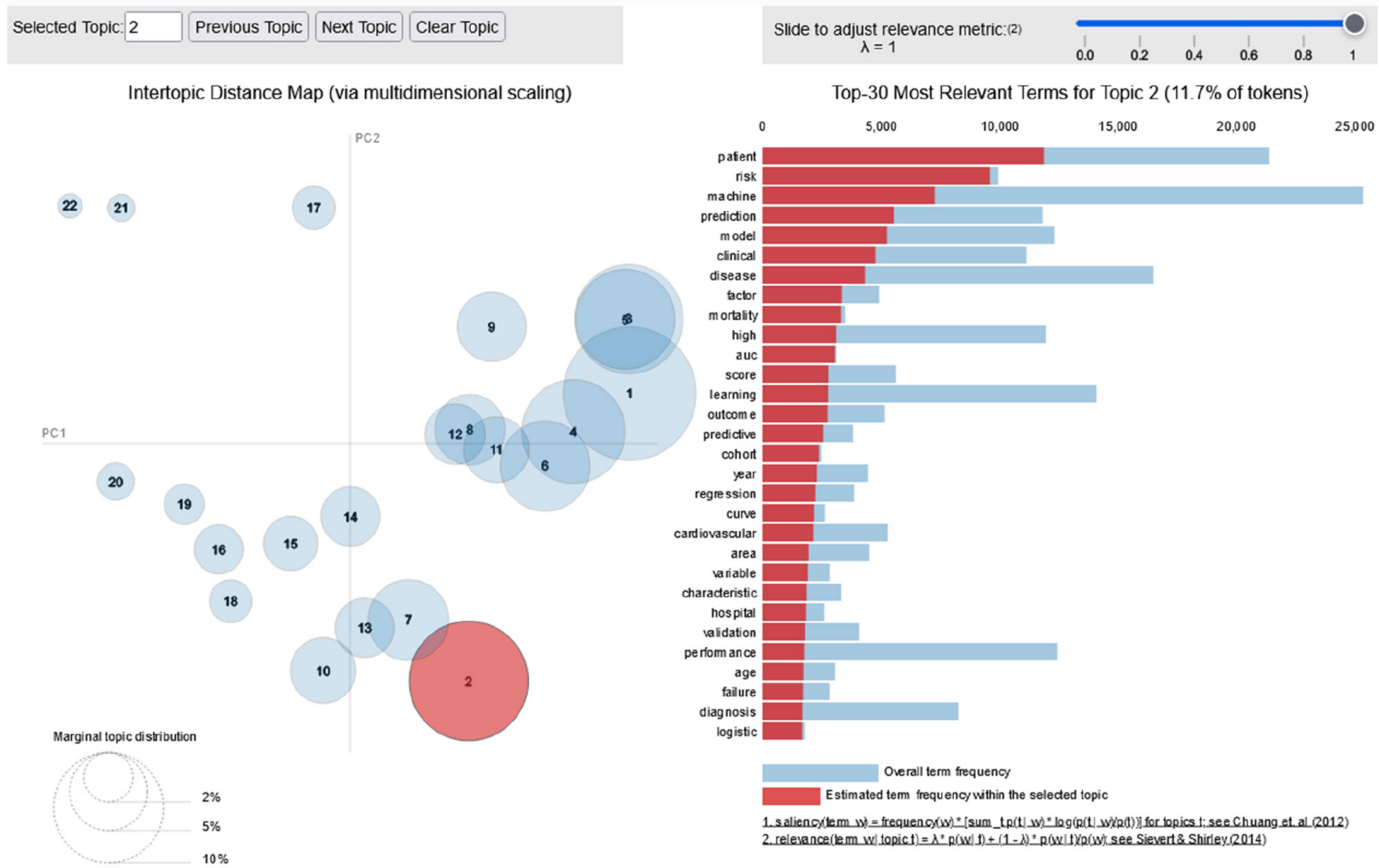
Inter-topic distance map of publications since 2010–2022. PC, principal component, the numbers inside the circles indicate number of topics and the size of circle indicate topic dominance.

The intertopic distance map is an interactive visualization of topics identified by the LDA model. It shows how the topics differ from each other (24). More specifically, the left panel of an intertopic distance map shows the global view of topic prevalence and how the topics relate to each other (by plotting topics as a circle). The right panel shows keywords contributing to the topic (by plotting each keyword as a bar chart based on their frequency) (24). Although we were able to identify 22 topics (Table 3), the result showed how some topics are closely related and some of them are distinct in the context of AI application in CVD (Figure 7). The provided intertopic distance map on Figure 7 offers a visual representation of the relationships and distances between different topics generated through topic modeling. By observing the intertopic distances on the map, we can discern patterns of topic similarity and divergence. Topics located closer to each other on the map are more closely related, sharing common keywords or themes. Conversely, topics positioned farther apart exhibit greater dissimilarity, suggesting unique subject matter or content.

**Table 3:** List of topics with AI application in cardiovascular disease in alphabetical order and their associated number of publications.

No.	Identified topics	Associated number of publications
1	Aneurysm and machine learning	362
2	Atheroscloretic plaque and machine learning	112
3	Biomarkers and protein/gene analysis using ML in CVD context	785
4	Blood pressure and machine learning	1,379
5	Cardiac image analysis	6,401
6	Cardiac motion estimation	292
7	Cardiovascular disease risk/outcome prediction	1,108
8	Clinical application of ML in CVD	161
9	Coronary artery disease and machine learning	1,528
10	Heart failure and ML	594
11	Innovative systems and heart diseases	590
12	Liver fibrosis, cardiovascular disease and machine learning	336
13	Machine learning and atrial fibrillation	588
14	Machine learning and clinical decision systems	176
15	Machine learning and heart disease diagnosis	607
16	Machine learning and surgical outcome prediction	390
17	Neural network and occlusion detection	854
18	NLP, electronic health record and CVD	72
19	Real-time cardiac related data and machine learning	429
20	Robotic-assisted cardiac surgery	1,444
21	Stroke and robotic rehabilitation therapy	1,707
22	Wearable Sensors and heart signal analysis	1,401

**Figure 8:**
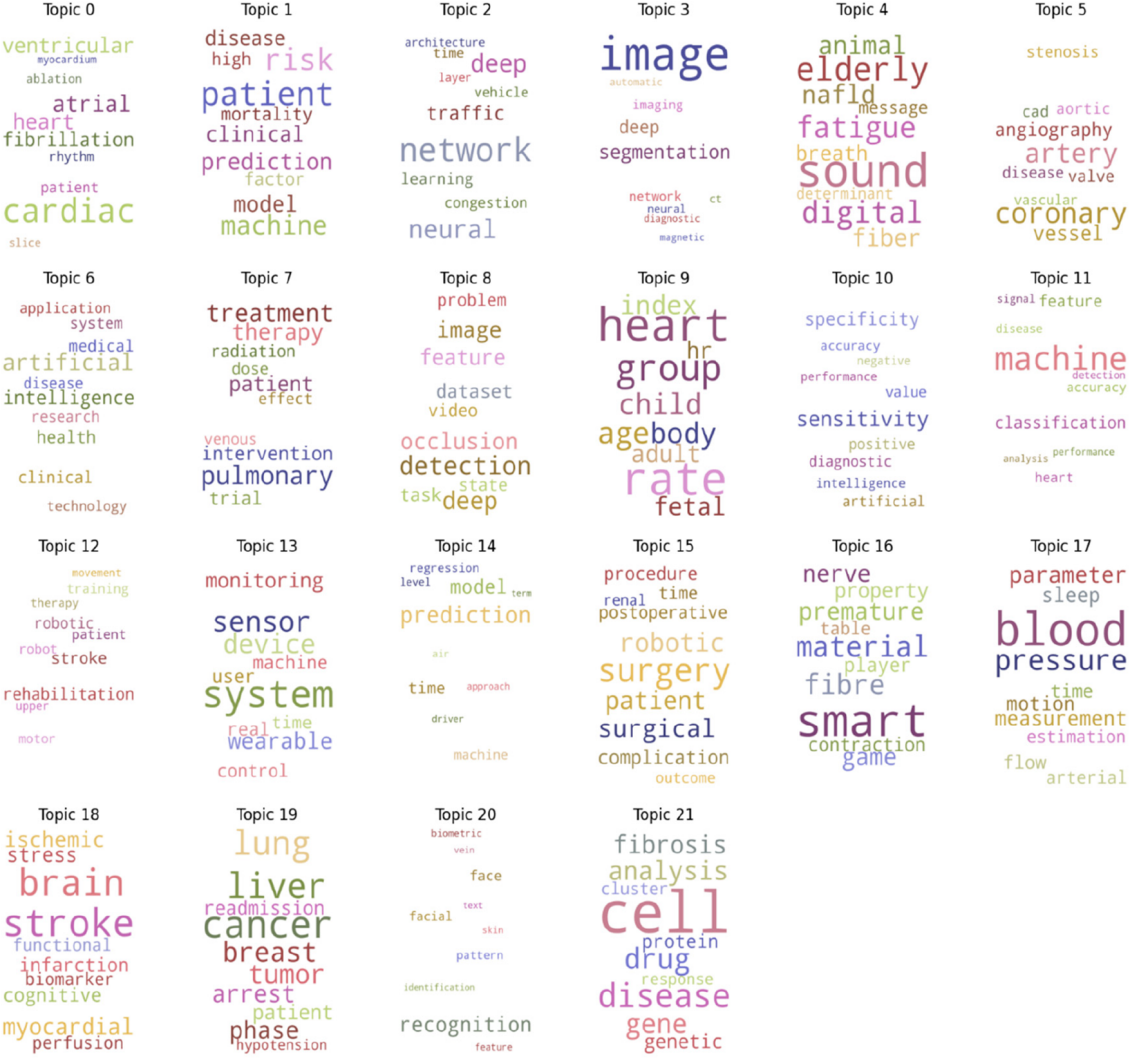
Word cloud of dominant topics in the context of AI application in CVD since 2000.

After identifying dominant topics, topic labeling was done based on the result from word relevance, inter-topic distance map and keyword weight. Afterward, the topic labeling was done by two experts (KBS and PW), it was further discussed among the working group (KBS, PW, AZ, DW) for consensus. The word cloud in Figure 8 highlights the relevant keywords for the identified topics. Prominent and larger words in the word cloud represent central concepts that recurrently emerge across multiple topics. These words can signify dominant subject areas or recurring terms that hold significance. In contrast, smaller words in the word cloud may represent less frequent or more specialized terms. These terms offer insight into nuanced or distinctive subtopics within the dataset. The word cloud's arrangement does not only highlight the most common words but also visually groups related terms together in terms of semantic distance. This visualization aids in identifying potential clusters of themes and understanding the overall content landscape.

**Figure 9:**
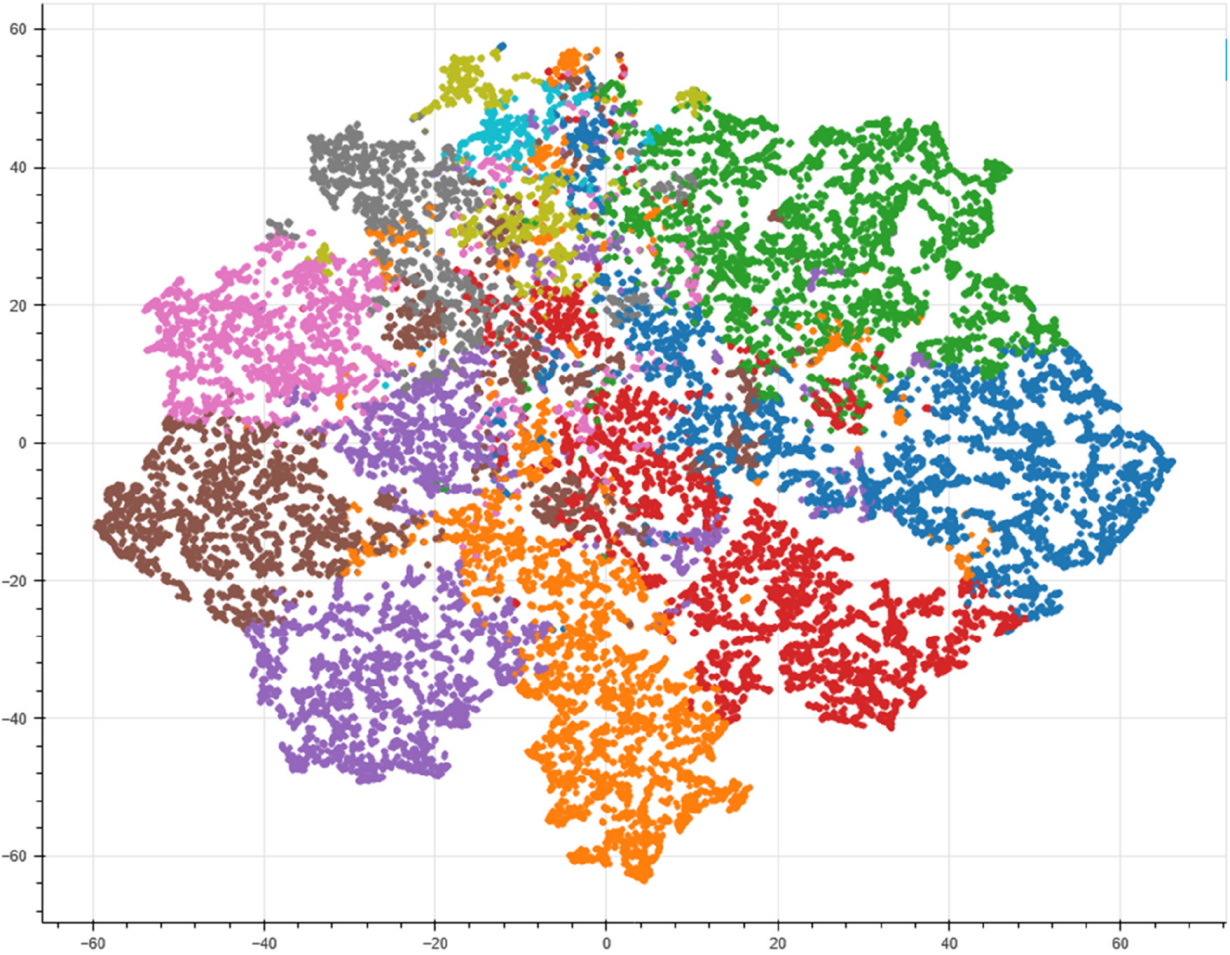
Cluster of topics in the application of AI in CVD since 2000. Each data points indicates publications and similarity in color shows the presence of common keyword and topic similarity. From here, cardiac image analysis, stroke and robotic rehabilitation therapy, coronary artery disease and machine learning, robotic-assisted cardiac surgery, wearable sensors and heart signal analysis, blood pressure and machine learning, and cardiovascular disease risk/outcome prediction are the predominant topics.

To get more insight, each publication was plotted as a datapoint in a 2-dimensional plot and clustered by the dominant topics using t-stochastic neighbor embedding (t-SNE) (Figure 9). The result showed that some topics are more dominant than the others such as cardiac image analysis and cardiovascular risk/outcome prediction (See the details of the identified topics in Table 3). In Figure 9, the clusters of topics that appear close together indicate high similarity (similar colours) in their distributions of words and themes. This suggests that these topics share common keywords or concepts and are likely to be related in content.

## Identified topics

We were able to identify 22 topics with AI application in CVD (Table 3). Our analysis shows an increase in number and diversity of topics over the years. Some topics like “stroke and robotic rehabilitation therapy” and “Cardiac image analysis” persisted as major topics throughout the years. Other topics like “Retinal image analysis and CVD” emerge as a dominant new topic since 2020. Furthermore, the type and number of cardiovascular disease conditions in which AI is becoming a major methodical solution increase over time. Diseases like “coronary artery disease”, “Myocardial infraction/Heart attack” and “stroke” are the widely studied contexts. The results also indicate that prediction models for prognosis/diagnosis of heart disease are becoming more prevalent. Furthermore, image and signal analysis are the most widely applied application of AI in CVD context.

**Figure 10:**
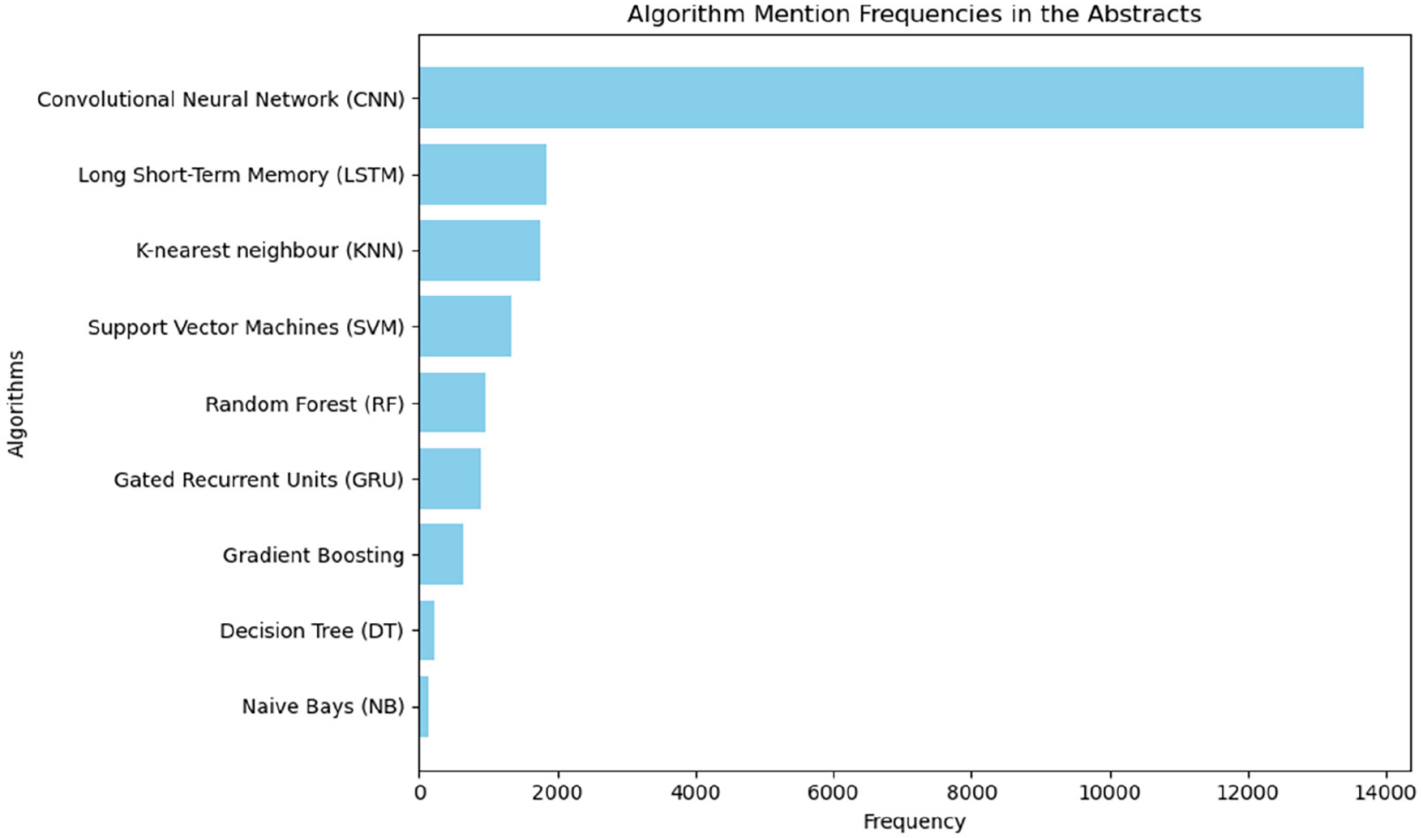
Algorithmic mentions in abstracts of studies on AI-CVD studies since 2010–2022. This marks the relevance of neural network architecture in cardiovascular medicine (CNN, LSTM, GRU).

In this rapidly evolving landscape of scientific research, the application of advanced algorithms has become pivotal. To get the grasp in the relevant algorithms used in AI related cardiovascular research, we delve into the mention and utilization of algorithms within the abstracts of our study's corpus. Through a meticulous analysis, we aim to illuminate the prevalence, diversity, and significance of algorithms as integral tools shaping contemporary research paradigms. We listed all the available supervised, unsupervised and reinforcement learning algorithms, NLP algorithms, and deep learning algorithms and calculated the number of mentions in the abstracts. This will give us a glimpse of information which algorithms are widely used in cardiovascular research. The number of mentions is not a certain measure that the studies used may not necessarily ascertain the use of these algorithms but can show the prevalence of discussion about the algorithms in the abstracts which gives an idea of the algorithms that are being talked about. Figure 10 shows that CNN (Convolutional Neural Network) is the most mentioned algorithm among the abstracts followed by LSTM (Long Short-Term Memory) and KNN (K-nearest Neighbour). From the result, we can see that neural network architectures are widely used in AI-CVD studies which makes sense given that the most dominant area of research identified was cardiac image analysis.

**Figure 11:**
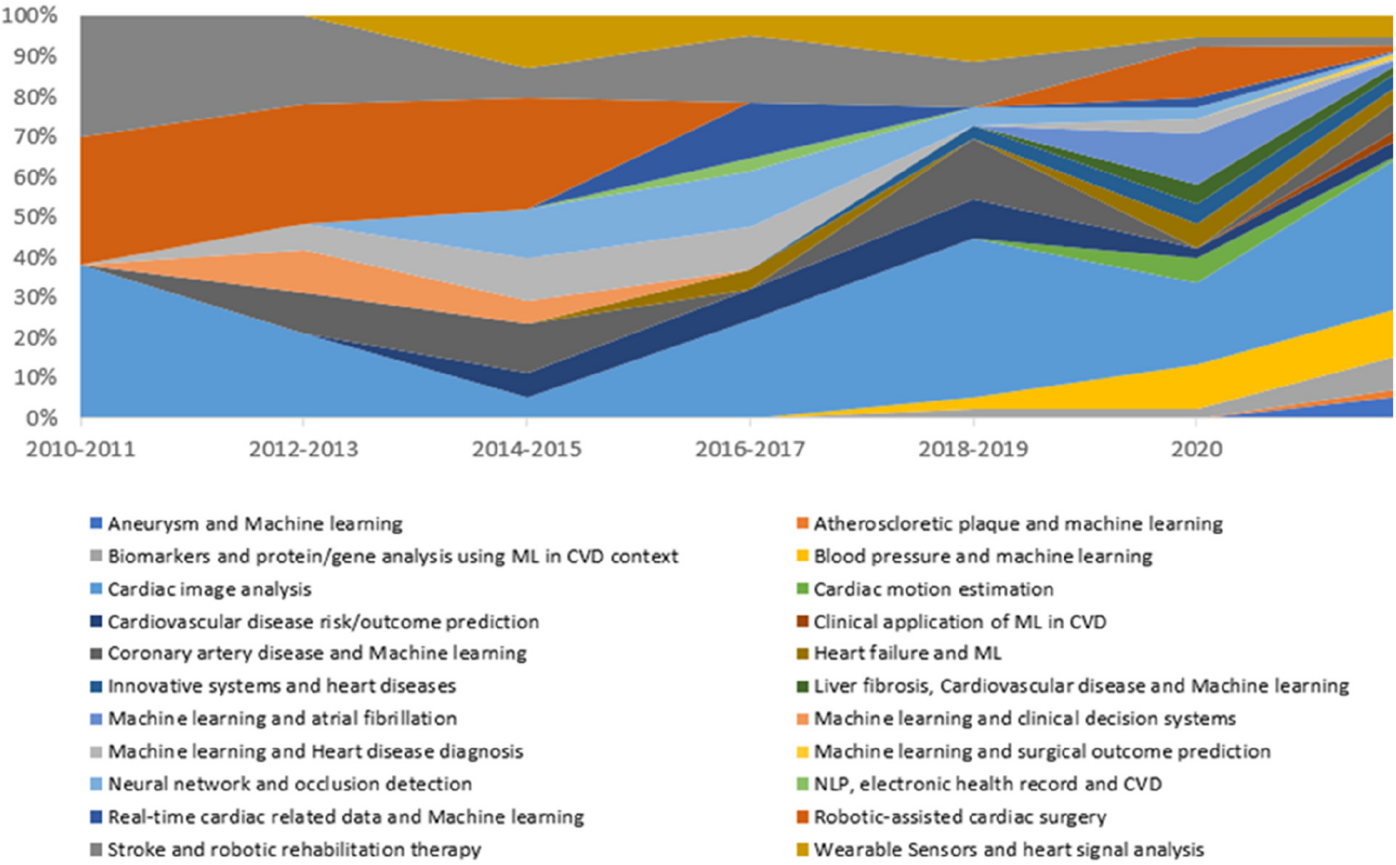
Topic change over time from 2010 to 2022 in the context of cardiovascular disease and AI application.

Identifying the topics and most mentioned algorithms provide an interesting information but to see the evolution of these topics over time is also important. The topic change over time (Figure 11) shows that the number of publications and diversity of topics significantly increased over the years. In 2010 and 2011, there were fewer number of topics (the studies were mainly focused on three topics (stroke and robotic rehabilitation, cardiac image analysis and robotic assisted cardiac surgery) and that changed over the years and yet cardiac image analysis is still the widely studied topic in the context of AI in CVD.

## Discussion

In this work, we applied bibliometric analysis and topic modeling based on publications indexed in Web of Science and PubMed to evaluate research trajectories and to identify AI topics focused on cardiovascular disease.

The result showed a substantial increase in publications since 2016. This trend is not specific to AI applications in CVD but it can be observed in most AI studies in medical context (25, 26). Aside from the enormous increase in publication, studies showed a significant geographical disparity in contribution and collaboration globally (Figure 4). This might result in an “AI divide”, as reported by Lutz et al. indicating that most of the contributions and networking are cantered in developed countries (27). AI divide refers to the gap in level of access, usage and concern for AI technologies between demographic regions of the world. According to the literature, this gap is widening rather than closing (7, 27). This observation is backed up by our reported result. Potential reasons include socio-economic factors, the fact that AI studies are usually computer power intensive, and the gap in research and data infrastructure (28–30).

We also observed that the majority of publications associated with AI and CVD are indexed in WoS and CVD is the widely studied context compared to other conditions. The contribution in publication from the USA and China was identified as the major without normalizing to the population size. However, the multiple country collaboration (MCP) was higher in UK, German and Australia with 42.8%, 40.3% and 40.0% respectively (Table 2). This finding was in line with other studies showing more networking among European countries (26).

The distribution of word counts in the abstract showed a composition of abstracts with different number of words ranging from 50 to 500 words with mean word distribution of 215 words (Figure 6). Which was considered good fit given the fact that most journals use 350 words as a limit. The number of topics were identified by using coherence score and resulted in *k* = 22 (*k* = number of topics). The intertopic distance map showed the relationship between the 22 topics signifying that some topics were closely related and some of them were distinct (Figure 7). The most important keywords responsible for each topic were visualised using wordcloud (8) and the 2-dimentional t-SNE plot showed the topic distribution by taking each abstract as a data point and colour them based on topic similarity (Figure 9).

We identified 22 general topics from the corpus of 23,846 abstracts. One of the extensively explored aspects of AI applications in CVD is “cardiac image analysis”. Cardiac Image ranges from 3D/4D of Magnetic Resonance Images (MRIs), echocardiography (ECG), Ultrasound images, Computed Tomography (CT) scans and Nuclear medicine imaging. The analysis includes image segmentation, classification and pattern recognition. Furthermore, the application of neural networks and multiple deep learning algorithms in cardiovascular image analysis has become an outstanding solution over the years (31). Studies pined that deep learning has revolutionized computer vision in CVD imaging and that has a remarkable potential to facilitate diagnosis and prognosis. This finding contends with a similar study conducted by Tran et al., denoting that robotic assisted surgery and stroke rehabilitation were the dominant topics (7). Our results, however, indicated an upsurge of research focus on cardiac image analysis. The differing observations could be explained by the difference in study periods and context differences. The former study compiled search results from 1991 to 2018 and focused on the context of stroke and heart disease whereas we gathered publications from 2000 to 2022 and focused on the general cardiovascular disease groups. In other word, the former study included 18 years of publications that we didn't include (1991–2009) which might contribute to the difference in keyword relevance/weight. Conversely, our research incorporated data from the recent 3 years (2019–2022) representing a significant portion of the overall publications, which the prior study did not include.

Over the past decade, “Robotic-assisted cardiac surgery” has consistently emerged as the second most dominant topic of study. Robotic assisted cardiac surgery is a cardiac surgical procedure assisted by robotic technologies with minimal invasive techniques (32). Frequently studied cardiac cases include mitral valve repair, cardiac revascularization, left ventricular lead placement, aortic valve repairs, cardiac resynchronization therapy (CRT), tumour resections, endoscopic coronary artery bypass grafting, atrial fibrillation ablation and cardiac arrhythmia surgery (33). Surgical robotic systems are certainly one important element in these procedures, including the widely known surgical systems “The DaVinci surgical system (Intuitive Surgical, Inc.; Sunnyvale, Calif)” and The ZEUS system (34, 35).

The topic of “Stroke and robotic rehabilitation therapy” was identified as a third dominant topic. Considering its importance for patients with motor disorder, the number of studies in rehabilitation robotics has also profoundly increased over the years (36). Due to the motor disfuntioning effect of Stroke, rehabilitation therapy is considered almost always to recover motor disorder in patients with Stroke. The potential of robotic devices in facilitating rehabilitation in stroke patients is one of the main research topics (37).

Apart from the persistent topics already discussed in the literature, new topics such as “biomarker” and “wearable sensor signal analysis” are gaining more attention. Biomarker is an objectively measured attribute considered as an indicator of normal biological, pathogenic or pharmacologic reaction to therapeutic intervention (38). The use of circulating, genetic and imaging biomarkers in cardiovascular risk prediction, diagnosis and prognosis has gained considerable attention (39). Accordingly, AI models are applied to identify and analyse most relevant cardiovascular biomarkers in diagnostic or prognostic performance. These novel methods are another step towards precision and personalized medicine (40).

The algorithmic mention graph showed that neural network architectures are the widely used method in cardiovascular AI research with most of its application in cardiac image analysis. This is inline with the review conducted by Litjens et al. (31). Convolutional neural network appears to be the most mentioned algorithm followed by LSTM and KNN. This indicates that the future direction of AI cardiovascular research is predominantly neural network and image analysis.

Overall, the application of AI in cardiovascular medicine has grown both with respect to scientific production and dimensions of topics. Topics and trends change with technological advances and improvement of methodological approaches. Thus, the mapping and modeling of topics over time is an important element in directing research and future guideline development.

## Limitations

Although we were able to identify the predominant topics in AI-related cardiovascular studies, one limitation of topic modeling is the subjective judgment on topic labelling. The algorithm can only suggest keywords based on probabilistic statistical computations. The actual labelling of topics is the researchers’ task. We used different techniques to identify the most appropriate topic labels. First, we labelled the topics independently with two co-authors based on the identified keywords. We then performed a crosscheck with the suggestion of the other two co-authors and amended based on consensus. We believe that the multidisciplinary background of co-authors helped in handling this challenge.

A notable limitation of our study is the challenge we faced in labelling certain topics as it was difficult to correlate the keywords with a cardiovascular context. Furthermore, our analysis was confined to English-language studies and studies indexed in WoS and PubMed which potentially omits pertinent studies published in other languages and studies that are not indexed in Scopus or PubMed.

## Conclusion

In light of the overall growth in scientific contributions in AI-related cardiovascular research, it's evident that the significant imbalance in collaboration across world regions remains a notable challenge, exacerbating the AI divide between developed and developing countries. Alternative solutions like open data and platform for sharing AI research infrastructures should be considered to attain regional and international partnership on knowledge sharing, access to science, technology and innovation.

Our analysis indicated a growing interest and diversity of topics over the years. Cardiac image analysis, robotic assisted cardiac surgery and stroke and robotic rehabilitation therapy are among the predominant topics identified. The result also showed that neural network architectures mainly convolutional neural network, LSTM (Short Long-Term Memory) and GRU (Gated Recurrent Unit) followed by KNN (K-Nearest Neighbour), SVM (Support Vector Machines) and RF (Random Forest) machine learning algorithms. Furthermore, topics such as biomarker and wearable signal analysis are the emerging dominant topics. Detailed refining research focused on the identified topics is required to explore the specifics of AI functionalities in cardiovascular medicine.

## Data Availability

The raw data supporting the conclusions of this article will be made available by the authors, without undue reservation.
